# B-1a Cell Development in Splenectomized Neonatal Mice

**DOI:** 10.3389/fimmu.2018.01738

**Published:** 2018-07-30

**Authors:** Gabriel K. Pedersen, Xiaohong Li, Sharesta Khoenkhoen, Monika Ádori, Bruce Beutler, Gunilla B. Karlsson Hedestam

**Affiliations:** ^1^Department of Microbiology, Tumor and Cell Biology, Karolinska Institutet, Stockholm, Sweden; ^2^UT Southwestern Medical Center, Center for the Genetics of Host Defense, Dallas, TX, United States

**Keywords:** B-1 cells, transitional B cells, splenectomy, B cell progenitors, fetal liver

## Abstract

B-1a cells are mainly generated from fetal liver progenitor cells, peri- and neonatally. The developmental steps and anatomical sites required for these cells to become mature B-1a cells remain elusive. We recently described a phenotypically distinct transitional B cell subset in the spleen of neonatal mice that generated B-1a cells when adoptively transferred. This, in combination with findings demonstrating that B-1a cells are lacking in congenitally asplenic mice, led us to hypothesize that the neonatal spleen is required for B-1a cell development. In accordance with previous reports, we found that B-1a cell numbers were reduced in adult mice that had undergone splenectomy compared to after sham surgery. In contrast, neonatal splenectomy led to peritoneal B-1a cell frequencies comparable to those observed in sham-operated mice until 6 weeks after surgery, suggesting that an intact spleen is required for B-1a cell maintenance rather than development. To study the role of the prenatal spleen in generating B-1a cells, we transferred fetal liver cells from pre-splenic embryos [embryonic age 11 (E11) days] into splenectomized recipient mice. B-1a cells were generated in the absence of the spleen, albeit at slightly reduced frequencies, and populated the peritoneal cavity and bone marrow. Lower bone marrow B-1a cell frequencies were also observed both after neonatal and adult splenectomy. These results demonstrated that B-1a cells could be generated in the complete absence of an intact spleen, but that asplenia led to a decline in these cells, suggesting a role of the spleen for maintaining the B-1a compartment.

## Introduction

Congenital asplenia, or splenectomy, leads to increased susceptibility to infections with encapsulated bacteria such as *Haemophilus influenzae* and *Streptococcus pneumoniae* (1, 2), but the causes for this are poorly understood. Antibody responses to the polysaccharide capsule [T-independent type 2 (TI-2) antigens] on the surface of these bacteria are reduced in the absence of spleen (3–5) and lack of certain B cell subsets important in the early anti-bacterial response, including marginal zone B (MZB) cells, may be one reason for this (6). In addition, B-1a cells protect against encapsulated bacteria by constitutively secreting broadly reactive natural IgM antibodies (7, 8) and it was reported that removal of the spleen in adult mice leads to reduced B-1a cell frequencies, demonstrating that the spleen is either required for maintenance and/or for development of B-1a cells (3, 9–11). Further indications that the spleen is required for B-1a cell development came from analysis of mice with congenital asplenia due to absence of the *Tlx1* (*Hox11*) gene since, in these mice, B-1a cells were essentially absent. The underlying mechanisms for the lack of B-1a cells under asplenic conditions, however, remain unknown (3).

B-1a cells play non-redundant roles for early protection against a number of pathogens (7, 12). B-1a cells participate in the antibody response against T-independent antigens (7, 13) and secrete poly-reactive natural IgM antibodies with protective and immune homeostasis functions (6, 13, 14). B-1a cells, through production of IL-10 and other immunosuppressive mediators also have important roles in immune regulation and in ameliorating inflammatory diseases (15, 16). The peritoneal cavity is the main reservoir for B-1a cells, in which they comprise approximately 50% of B cells (17). B-1a cells are also located systemically and in spleen and bone marrow, although at lower frequencies (0.5–2% of B cells) (14). In contrast to B-2 cells, which are continually replenished from hematopoietic stem cells in the bone marrow, B-1a cells predominantly develop during fetal and neonatal life and are maintained by self-renewal (18). Thus, early yolk sac, splanchnopleura, and fetal liver mainly give rise to B-1a rather than B-2 lineage cells (18, 19).

Developing B cells migrate to the spleen to undergo transitional T1 and T2 stages (20). For B-1a cells, the transitional window is limited to early life and we recently demonstrated that TrB-1a cells are a distinct subset of transitional B cells in neonates (21). This identification of TrB-1a in the neonatal spleen led us to ask whether the neonatal spleen is required for B-1 cell development. To address this, we splenectomized neonatal wild-type (wt) mice (neo-splx) and examined B cell development. Compared to sham-operated mice, the neo-splx mice displayed a rapid twofold reduction in bone marrow and blood B-1a cells, but peritoneal B-1a cell frequencies were normal until 6 weeks after surgery. To investigate if the fetal spleen is required for B-1a cell development, we transferred wt fetal liver (FL) cells into splenectomized or sham-operated RAG1−/− mice. For the transfer we used FL from embryonic age 11 days (E11), thus prior to the point in development where the spleen primordium is developed, which in mice occurs at E12.5–13. The mice that had undergone splenectomy in conjunction with FL transfer had high frequencies of donor-derived B-1a cells, demonstrating that cells of B-1a phenotype may develop under completely asplenic conditions. Interestingly, asplenic conditions, both when modeled from prenatal, neonatal, and adult stages led to a reduction in bone marrow B-1a cells, which may imply distinct developmental or homeostatic requirements for B-1a cells in different compartments.

## Materials and Methods

### Mice and Surgery

Mice were maintained in the animal research facility at the Department of Microbiology, Tumor and Cell Biology (MTC), Karolinska Institutet, or at the University of Texas Southwestern Medical Center. Studies were performed in accordance with institutionally approved protocols or with Committee for Animal Ethics (Stockholms Norra djurförsöksetiska nämnd) approval or Institutional Animal Care and Use Committee approval. C57BL/6J mice were purchased from the Jackson laboratory, and *Rag1*−/− mice, on C57BL/6J background, were bred at MTC. Mice harboring the *bumble* mutation were described previously (21, 22). Adult wt or *Rag1*−/− C57BL/6J mice were anesthetized by isofluorane *via* a nose cone and shaved. A small incision was made in the skin at the left flank right above the spleen. The spleen was removed and the splenic arteries and venous supply carefully cauterized. The incision was closed with surgical silk-thread (Ethicon) and buprenorphine analgesia was administered. For neonatal splenectomy, ice was used as anesthetic. Sham-operated mice underwent the same procedure as splenectomized mice, except removal of the spleen.

### Cell Preparation

Splenocytes and fetal liver cells were prepared as a single cell suspension using a 70 µm cell strainer. Peritoneal cells were isolated by flushing with cold PBS/1% FBS (1–10 ml, dependent on mouse age). Peritoneal cells were discarded if contaminated with blood. Femurs and tibias were flushed with a 26G needle. Cell suspensions were diluted in RPMI-1640 supplemented with 2 mM l-glutamine, penicillin (100 IU)–streptomycin (100 µg/ml), 5 × 10^−5^M β-mercaptoethanol (Gibco), and 10% fetal bovine serum (complete RPMI). Splenocyte and bone marrow cell suspensions were washed once in Ca^2+^- and Mg^2+^-free PBS and treated with red blood cell lysis buffer before further processing. For reconstituting B-1 cells in splenectomized or sham-operated *Rag*−/− mice, total embryonic age 11 days fetal liver cells (1.5 × 10^6^ cells) were prepared and transferred intravenously (i.v.).

### Immunization

Mice were immunized with 50 µg NP (40)-Ficoll (Biosearch technologies) diluted in PBS and 200 µl was injected intraperitoneally (i.p.).

### ELISA

ELISA was performed by coating ELISA plates (Nunc) with unconjugated anti-mouse IgM (Southern Biotech). After incubation overnight (4°C), washing (PBS + 2% Tween20) and blocking for 1 h with PBS containing 2% dry milk (blocking buffer), serum was added in threefold serial dilutions in blocking buffer and incubated for 1.5 h at room temperature (RT), before addition of HRP-coupled anti-IgM or IgG3 (Southern Biotech). The assay was developed with 3,3′,5,5′-tetramethylbenzidine (TMB) substrate (KPL) followed by 1M H_2_SO_4_ and the OD values were read at 450 nm using an Asys Expert 96 ELISA reader (Biochrom).

### Enzyme-Linked Immunosorbent Spot (ELISpot)

Detection of total IgM-producing cells from bone marrow was performed using an ELISpot assay. MultiScreen-IP filter plates (Millipore, MAIPSWU10) were pre-treated with 70% ethanol and washed in sterile PBS. Plates were coated with 5 µg/ml anti mouse IgM (Southern Biotech) in PBS and incubated overnight at 4°C. The following day, plates were blocked in complete RPMI medium with 50 µM 2-mercaptoethanol and 10 mM HEPES for 1 h at 37°C and 2.5 × 10^5^ cells added. Plates were incubated for 16 h at 37°C in 5% CO_2_. The plates were washed and 0.1 μg/well of biotinylated anti-mouse IgM (Mabtech) diluted in PBS was added to the wells. After 2 h of incubation at RT, plates were washed and steptavidin-ALP (Mabtech) added for 1 h, before the plates were developed with 100 µl of 5-bromo-4-chloro-3-indolyl phosphate/nitroblue tetrazolium chloride (BCIP/NBT)-plus substrate (Mabtech). The reaction was stopped by tap water. Spots were counted in an ELISpot reader (CTL).

### Flow Cytometry

Cells were incubated with Fc block (anti-CD16/32, BD) and stained with different panels of fluorochrome-conjugated monoclonal antibodies in PBS/2% FBS using the following antibodies: CD5 BV421 (S3-7.3), CD19 PE (1D3), CD19 FITC (1D3), CD19 BUV395 (1D3), CD43 APC (S7), B220 AF-700 (RA3-6B2), B220 PerCP, CD11b BV605 (M1/70) (all BD), CD93 APC (AA4.1), CD93 PE (AA4.1), B220 APC-EF780 (RA3-6B2), IgM eFlour450 (II/41) (all eBioscience), CD5 biotin (Biolegend), and IgM FITC (Fab_2_, polyclonal) (Southern Biotech). In some antibody panels, primary staining was followed by addition of streptavidin-AF488 (Life technologies). Samples were run using a BD LSRFortessa or LSR II and data were analyzed in Flowjo v9.6.4 (Treestar).

### Statistics

Differences between groups were analyzed by a Student’s *t*-test (GraphPad Prism v5.0 or 6.0f).

## Results

### The perinatal Spleen Is a Major Organ for B-1a Lineage Cells

**Figure 1:**
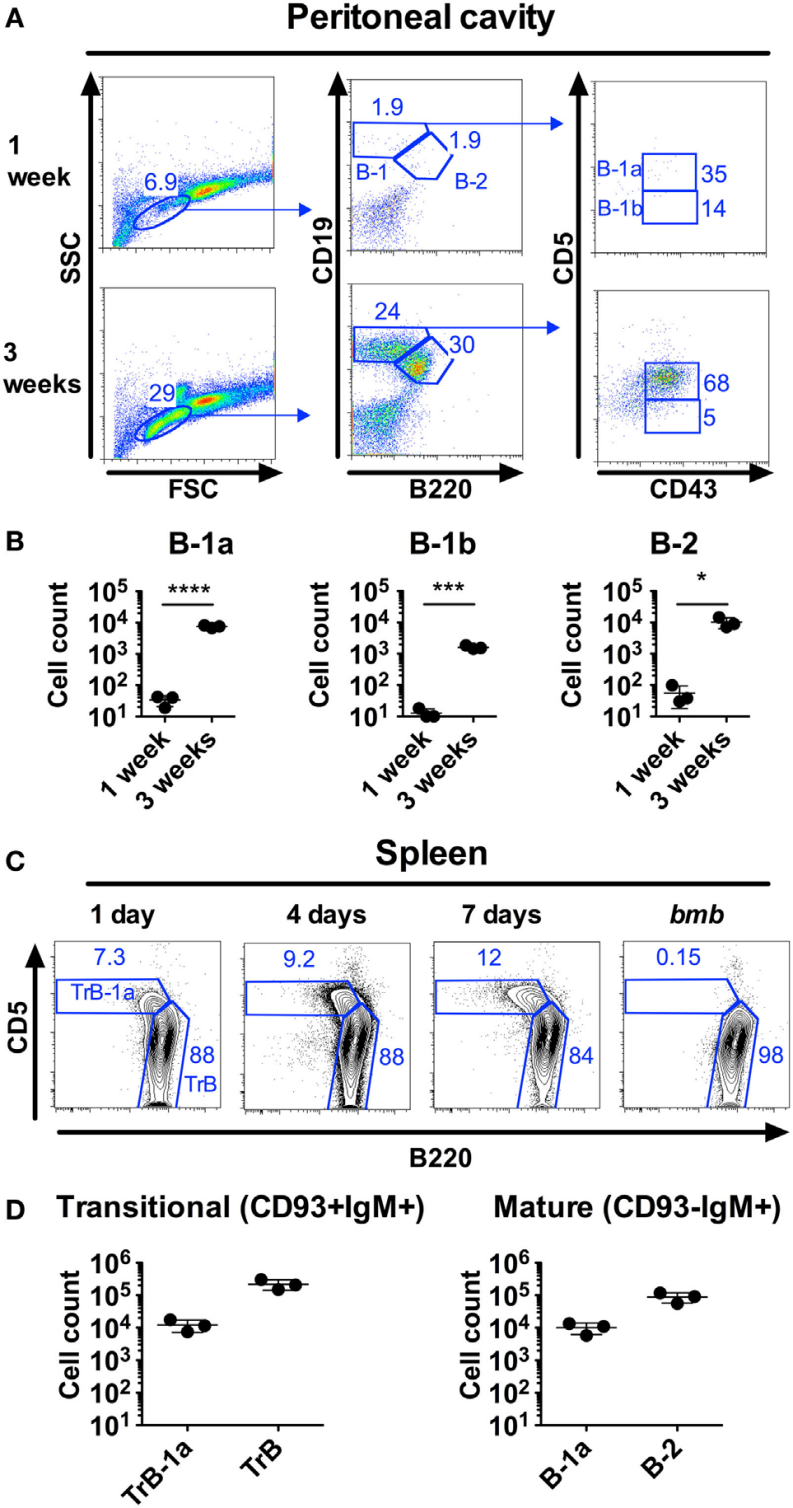
B-1 cells are found in the spleen but not in the peritoneal cavity of neonatal mice. Neonatal mice were stained by flow cytometry for B-1 and B-2 cells, defined as CD19hiB220loCD43+CD5+/− and CD19+B220+, respectively. **(A)** Representative staining of C57BL/6 wild type (wt) peritoneal cells from 1 to 3 weeks old mice. **(B)** Number of cells from the different B cell subsets in peritoneal flushes from 1 week to 3 weeks old wt mice. **(C)** Representative staining of splenic transitional B-1a (TrB-1a, CD93+IgM+CD5+B220lo) and transitional B (TrB, CD93+IgM+CD5−B220+) cells at different time points. As negative controls for transitional B-1a cells, neonatal mice with the *bumble* (*bmb*) mutation in the gene encoding IκBNS were included [these were previously demonstrated to lack TrB-1a, (21)]. **(D)** TrB-1a, TrB cells (left panel), and mature B-1a and B-2 cells (right panel) in the spleen of 1-week-old wt mice. Results are representative of at least two independent experiments and graphs display mean ± SD. Statistically significant differences are indicated by *, ***, and **** denoting *p* < 0.05, *p* < 0.001, and *p* < 0.0001, respectively by unpaired *t*-test.

The developmental steps from fetal liver (FL) progenitors to mature B-1a cells are incompletely understood. The FL gives rise to B-1a cells, but IgM+CD43+CD5+B-1a lineage cells constitute very few of CD19+ cells in liver perinatally (0.12% at E19) (23), suggesting that the FL is not the main site for the final steps of B-1a cell maturation. The peritoneal cavity harbors the largest frequency of B-1a cells in young and adult mice but B-1a cells are lacking in the peritoneal cavity of neonatal mice, constituting on average <50 cells in 1-week-old compared to 8 × 10^3^ cells in 3-week-old wt mice (Figures 1A,B). We previously described that neonatal but not adult mice harbor a population of splenic TrB-1a cells, that co-express common transitional B cell surface proteins (IgM+CD93+CD19+) and B-1a cell markers (CD5+CD43+B220lo) (21). Interestingly, the TrB-1a cell population is present already in spleen from 1-day-old mice, where they comprise 7% of IgM+CD93+ splenic transitional B cells (Figure 1C), which is much earlier than B-1a cells are first observed in the peritoneal cavity. 1-week-old wt mice harbor approximately 1.2 × 10^4^ splenic transitional B-1a, in addition to 1.0 × 10^4^ mature B-1a cells (Figure 1D). Thus, immature B-1a lineage cells seed the spleen before the peritoneal cavity, suggesting that the perinatal/neonatal spleen constitutes a niche for B-1a cell maturation.

### Splenectomy of Adult Mice Leads to a Rapid Decline in B-1a Cells

**Figure 2:**
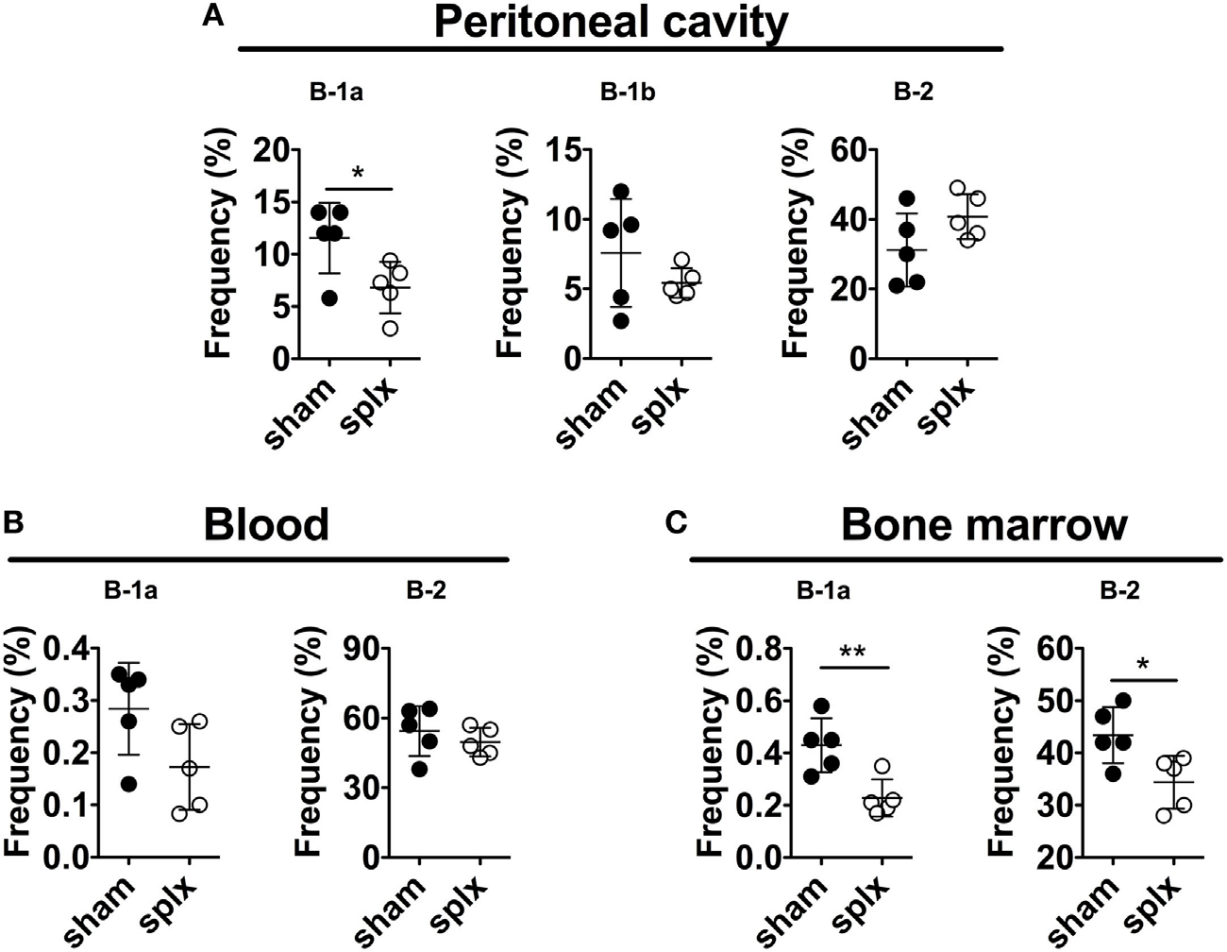
Splenectomy of adult mice leads to a decrease in B-1a cells. Adult 12-week-old wild-type mice were splenectomized or sham-operated and 10 days later stained for B-1 and B-2 cells (defined as in Figure 1) in **(A)** peritoneal cavity, **(B)** blood, and **(C)** bone marrow. The experiment was performed once and graphs display mean frequencies of the indicated cells in the lymphocyte gate ± SD. Statistically significant differences are indicated by * and **, denoting *p* < 0.05 and *p* < 0.01, respectively by unpaired *t*-test.

It was previously reported that splenectomy of adult mice leads to a rapid and persistent loss of peritoneal B-1a cells (3). We, therefore, examined the requirement of the adult spleen for B-1 cells defined as CD19hiB220loCD43+ and further sub-divided based on CD5 expression (CD5+B-1a and CD5−B-1b). Indeed, evaluation of B-1a cell frequencies 10 days after splenectomy revealed an approximately twofold drop in peritoneal B-1a cell frequencies compared to in sham-operated mice (statistically significant, *p* < 0.05) (Figure 2A). There was also a tendency toward lower numbers of peritoneal B-1a cells, although not statistically significant (*p* = 0.24) (Figure S1A in Supplementary Material). We observed no effect of splenectomy on peritoneal B-1b cells, while B-2 cell frequencies and numbers were slightly increased in splenectomized mice, although not statistically significant (*p* = 0.12). There was also a tendency toward lower blood B-1a cell frequencies and significantly lower bone marrow (BM) B-1a cell frequencies (*p* < 0.05, Figure 2C) and numbers (Figure S1B in Supplementary Material) in splenectomized compared to in sham-operated mice. BM B-2 cell frequencies were also lower in the splenectomized group (*p* < 0.05). Thus, adult splenectomy led to an approximately twofold drop in both bone marrow and peritoneal B-1a cell frequencies consistent with previous reports (3, 9, 11).

### The Neonatal Spleen Is Required for Maintaining Normal Frequencies of B-1a Cells but Is Not Required for Their Development

**Figure 3:**
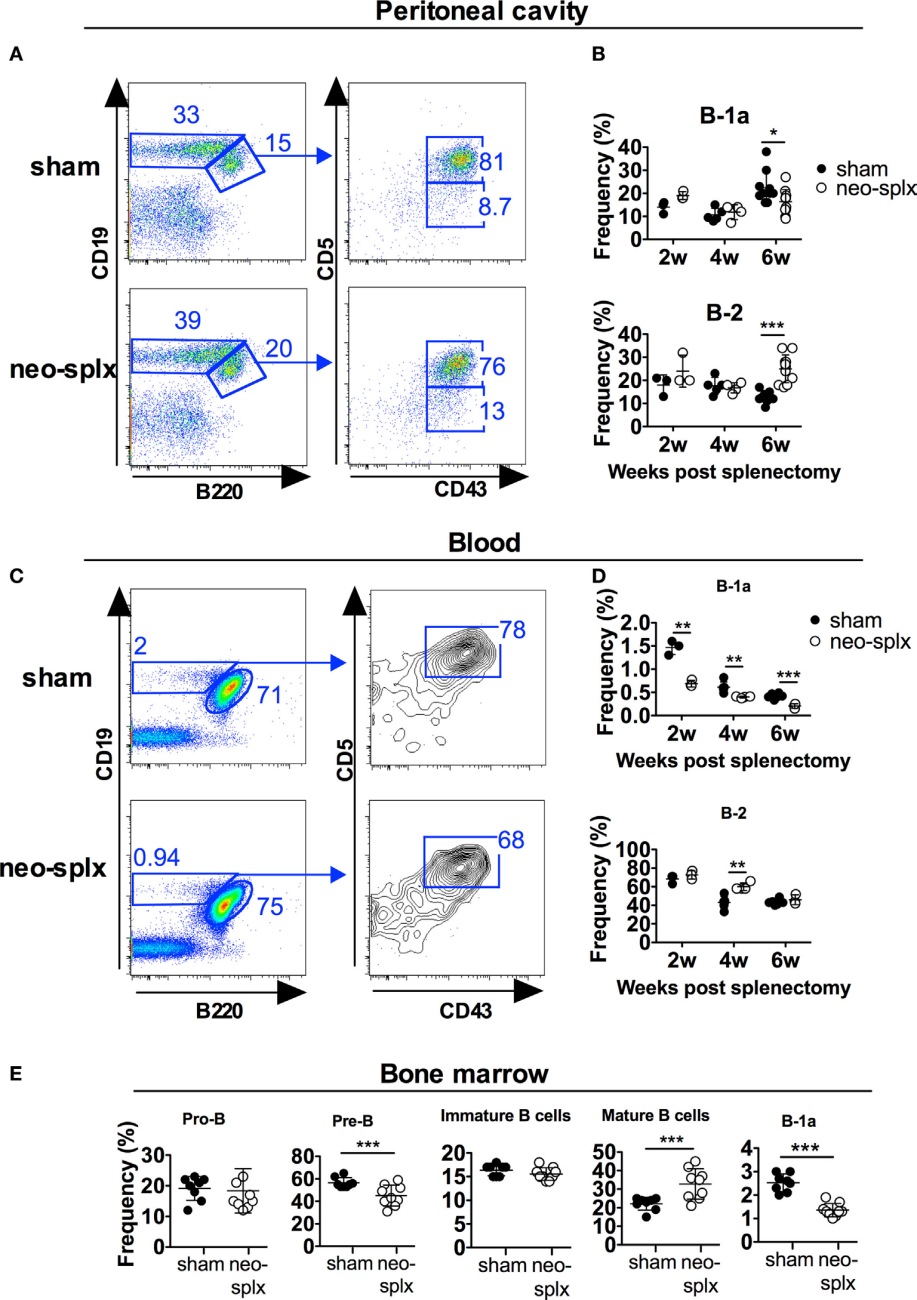
The neonatal spleen is not required for B-1a cell development but for maintaining normal B-1a cell frequencies. One-day-old wild-type pups underwent splenectomy (neo-splx) or sham surgery. Peritoneal B-1 cells were stained as defined as in Figure 1. **(A)** Representative staining of 4-week-old mice. **(B)** Peritoneal B-1a, B-1b, and B-2 frequencies at 2, 4, and 6 weeks after neonatal splenectomy. Statistically significant differences are indicated by * and ***, denoting *p* < 0.05 and *p* < 0.001, respectively by unpaired *t*-test. **(C)** Representative staining showing blood B-1a cells in 2-week-old mice. **(D)** Blood B-1a and B-2 cell frequencies at 2, 4, and 6 weeks after neonatal splenectomy. **(E)** Bone marrow B-lineage populations defined as B220+CD43+CD19+ (pro-B), B220+CD43−CD93+IgM− (pre-B), B220+CD43−CD93+IgM+ (immature B), B220+CD43−CD93−IgM+ (mature B), and CD93−CD19hiB220loCD43+CD5+ (B-1a) (Figure S3 in Supplementary Material). Results were pooled from several experiments with at least three mice per group assayed at each time point. Graphs display mean frequencies of the indicated cells in the lymphocyte gate ± SD. Statistically significant differences are indicated by *, **, and *** denoting *p* < 0.05, *p* < 0.01, and *p* < 0.001, respectively by unpaired *t*-test.

To examine if the neonatal spleen is required for B-1 cell development we performed splenectomy on 1-day-old mice and evaluated the frequencies of the different peritoneal B cell subsets at 2, 4, and 6 weeks thereafter. We observed no significant differences in peritoneal B-1a or B-2 cell frequencies at 2 or 4 weeks after neonatal splenectomy compared to after sham surgery (Figures 3A,B). Since the peritoneal cavity is not seeded with B-1a cells until after 1 week of age (Figure 1), and thus after the spleen was removed, these data indicate that the neonatal spleen is neither required for B-1a cell development nor for their migration into the peritoneal cavity. However, the neonatally splenectomized mice displayed reduced capacity to maintain peritoneal cavity B-1a cells since, at 6 weeks post splenectomy, the B-1a cell frequencies in the splenectomized mice were significantly lower (*p* < 0.05) than in the sham-operated mice. This reduction in B-1a cells was paralleled by increased B-2 cell frequencies (statistically significant, *p* < 0.001).

### Neonatal Splenectomy Leads to Reduced Blood and Bone Marrow B-1a Cell Frequencies

Contrary to that observed in the peritoneal washes, blood B-1a cell frequencies were twofold lower in neonatal splenectomized mice compared to in sham-operated littermates at all the time-points measured [2, 4, and 6 weeks after surgery (*p* < 0.01)] (Figures 3C,D). Neonatal splenectomy did not consistently affect B-2 cells in the blood, except at 4 weeks post-surgery, where splenectomized mice had significantly higher blood B-2 cell frequencies than mice that had undergone sham surgery (Figures 3C,D). To investigate if neonatal splenectomy had an impact on B cell progenitors, we examined these in the bone marrow 6 weeks after neonatal splenectomy (Figure 3E; Figure S2A in Supplementary Material). Compared to the sham-operated group, the splenectomized mice had significantly lower frequencies of B220+CD43−sIgM−CD93+ pre-B cells (*p* < 0.001) and increased B220+CD43−sIgM+CD93− mature B cells (*p* < 0.001), while immature B and pro-B cell frequencies were similar between the two groups. Approximately 2–3% of mature (CD93−) bone marrow B cells in 6-week-old wt mice displayed a CD19hiB220loCD5+B-1a cell phenotype. Notably, similar to in the blood, neonatal splenectomy led to an approximately twofold reduction in BM B-1a cells (1–1.7%) compared to the corresponding sham-operated littermate controls (*p* < 0.001) (Figure 3E; Figure S2B in Supplementary Material). In summary, the neonatal spleen was dispensable for peritoneal B-1a cell development, while circulating and bone marrow B-1a cell frequencies were reduced after neonatal splenectomy.

### B-1a Cells Develop in the Absence of Spleen Although at Reduced Frequencies

**Figure 4:**
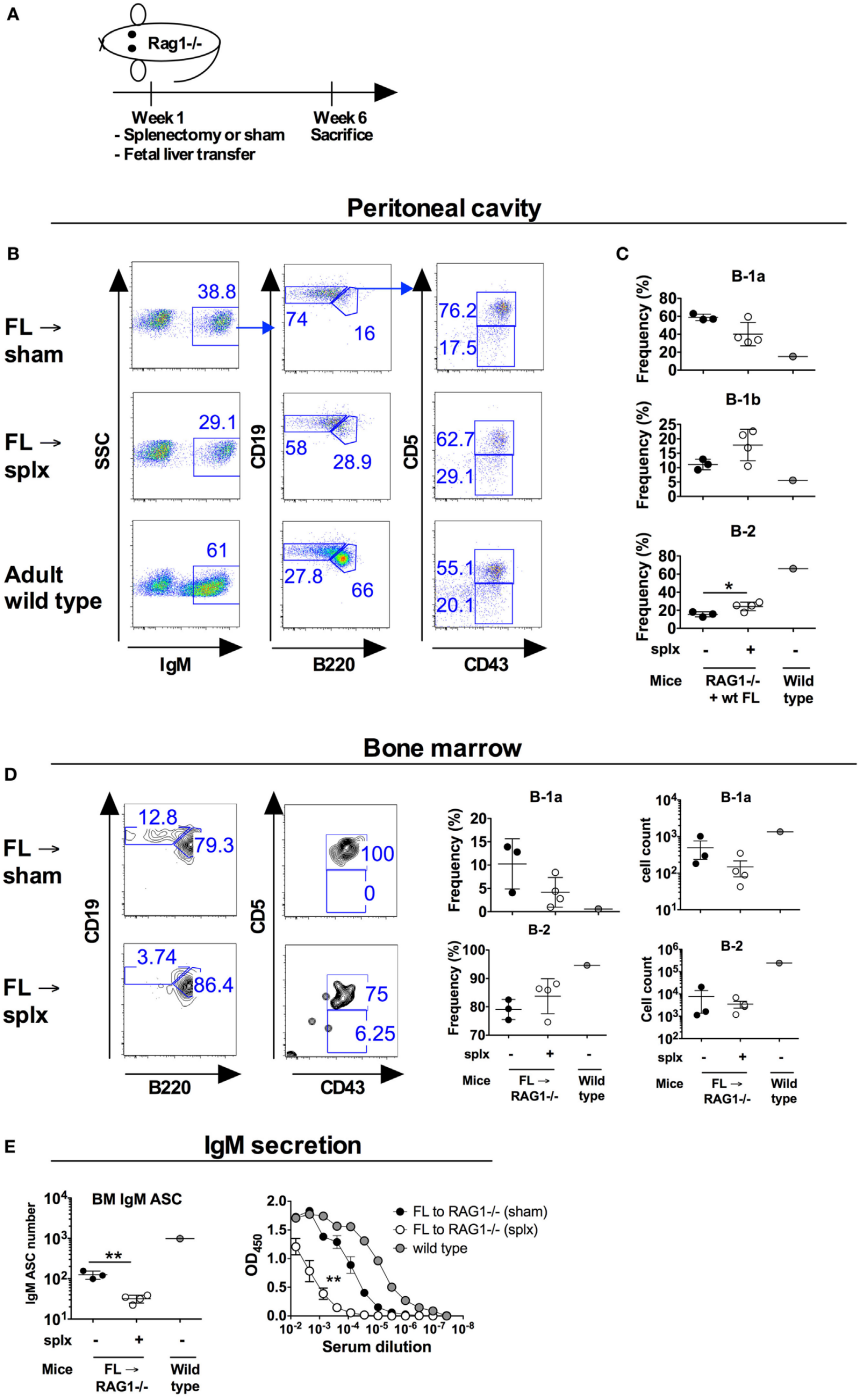
B-1a cell development from fetal liver B-1a progenitors transferred into conjointly splenectomized hosts. Wild-type (wt) fetal liver cells were isolated from 11 days old embryos (E11) and transferred to adult RAG1−/− mice that were subsequently immediately undergoing splenectomy or sham surgery. At 6 weeks later the mice were sacrificed and analyzed for B-1a cells. Donor-derived B-1a cells were identified as IgM+Cd19hiB220loCD43+CD5+. No IgM positive cells were observed in the peritoneum of RAG1−/− mice that had not received wt cells (not shown). **(A)** Schematic of the study. **(B)** Representative plots of peritoneal cavity B cells after transfer of E11 cells into splenectomized versus sham-operated recipients. **(C)** Frequency of the indicated B cell subsets in the peritoneal cavity of recipient mice as gated from IgM+ (donor-derived cells). **(D)** Representative plots and frequencies of bone marrow B-1a and B-2 cells after transfer of E11 cells into splenectomized versus sham-operated recipients. Plots were gated from IgM+ (donor-derived) cells. **(E)** Total bone marrow (BM) IgM antibody secreting cells depicted as total numbers from one femur (left panel) as measured by enzyme-linked immunosorbent spot and total serum IgM antibody levels as measured by ELISA. Sera were run in threefold dilutions with a starting dilution of 1:150 and statistics calculated by comparing the area under the curve (right panel). The experiment was performed once. Statistically significant differences are indicated by *, **, and ***, denoting *p* < 0.05, *p* < 0.01, and *p* < 0.001, respectively by unpaired *t*-test.

Our results so far suggested only a partial requirement for the neonatal spleen in maintaining normal B-1a cellularity. Previous studies using *Hox11*−*/*− mice as a model for congenital asplenia found that the spleen is strictly required for B-1a cell development (3). Taken together with the neonatal splenectomy results, we hypothesized that the spleen is required for B-1a cell development pre- rather than postnatally and investigated this possibility by using fetal liver cell transfer. It is known that E11 liver cells readily reconstitute B-1a cells, while the spleen primordium is only evident at E12 days (24) and is subsequently seeded by lymphocyte progenitors at E12.5–13. To examine B-1 cell development from fetal liver progenitors under asplenic conditions, we transferred “pre-splenic” E11 fetal liver cells into RAG1−/− mice that were subsequently immediately splenectomized. The reconstituted RAG1−/− mice were sacrificed 6 weeks later and examined for peritoneal B-1 cells (Figure 4A). Similar to that observed in previously reported fetal liver transfer studies, the vast majority of peritoneal B cells generated from E11 FL were of B-1a cell phenotype (18). Notably, RAG1−/− mice that had undergone splenectomy in conjunction with fetal liver cell transfer had a substantial fraction of peritoneal B-1a cells, although the frequency of these cells was lower than in sham-operated mice (not significant, *p* = 0.06) (Figures 4B,C). In adult wt mice, approximately 1–2% of bone marrow B cells are B-1a cells. In contrast, on average 10% of bone marrow B cells in sham-operated RAG1−/− mice that received wt FL displayed a B-1a phenotype, in accordance with the increased propensity for FL to give rise to B-1a cells (Figure 4D). Splenectomized FL recipient mice had lower reconstitution of bone marrow B-1a cells compared to sham-operated controls [2.5-fold drop in frequency to on average 4%, (not significant, *p* = 0.11)]. Mice that had undergone splenectomy conjointly with FL cell engraftment also had significantly lower levels of bone marrow cells spontaneously secreting IgM (*p* < 0.01) and of serum IgM antibodies (*p* < 0.01) (Figure 4E).

In a separate experiment we instead performed the splenectomy of RAG1−/− mice 30 days prior to the transfer of wt E11 FL. By this, the transferred cells would be in a milieu devoid of spleen-derived factors, which we speculated could otherwise have supported B-1a cell development. Similarly to when the spleen was removed in conjunction with FL engraftment, splenectomy 30 days prior to FL transfer gave rise to peritoneal and bone marrow B-1a cells, albeit at reduced frequencies, and lower serum IgM antibody levels than in the sham-operated mice, albeit this did not reach statistical significance (*p* = 0.09) (Figures S3A–E in Supplementary Material). Overall, these studies suggested that although B-1a cell frequencies were reduced upon asplenic conditions, the spleen was not absolutely required for B-1a cell development from FL progenitors.

## Discussion

The spleen is an important organ for eliciting immune responses to blood-borne antigens (24). The risk of sepsis is 10–20 times higher in splenectomized individuals than in the general population and children born with isolated asplenia often die during the first months of life from sepsis ascribed to bacterial infections at mucosal sites (25–27). Despite the clear involvement of the spleen in protecting against pathogens, it is still unknown if it is the lack of certain cell types, such as marginal zone macrophages, MZB, or B-1a cells, reduced levels of natural antibodies or other defects that lead to the increased susceptibility to infection observed in the absence of spleen (3, 28).

How B-1 cells develop and differentiate remains controversial. Putative B-1 progenitors can be seen in the FL from embryonic age 11 days (E11), but further differentiated B-1a (IgM+CD43+CD5+) cells are only observed at very low frequencies in the FL pre- and perinatally [0.12% at E19 (23)]. In neonatal 1-week-old C57BL/6 mice, peritoneal B-1a cells are rare. In contrast, B-1a lineage cells comprise a high frequency (approximately 10%) of splenic B cells in neonates and most of these are CD93+ immature/transitional [Figure 1 (21)]. This finding led us to investigate if the neonatal spleen is required for B-1a cell development by performing splenectomy on 1-day-old mice. We found that, compared to sham-operated littermate controls, neonatally splenectomized mice displayed similar peritoneal B-1a cell frequencies at 2–4 weeks post splenectomy. This finding was unexpected, since splenectomy of adult mice leads to a rapid reduction in peritoneal B-1a cell frequencies [(3, 9, 29), Figure 2A]. Based on these data, we speculate that the spleen has a role in maintenance of peritoneal B-1a cells, but in neonatal mice the splenectomy associated B-1a cell loss is masked by *de novo* B-1a cell development (23). Supporting this, we observed a significant reduction in peritoneal B-1a cells at 6-weeks post-neonatal splenectomy. B-1 cell generation wanes during neonatal life and, possibly, absence of spleen at or after 6-weeks-of-age leads to reduced B-1a cell frequencies, similar to that observed in adult mice.

Little is known about the development of the human spleen. Recently, haploinsufficiency for the RPSA gene encoding ribosomal protein SA was identified as one factor associated with isolated congenital asplenia (27). *Hox11* (*Tlx1*) was also implicated in human splenic development (30). Mice lacking the *Hox11* gene are born asplenic, without other detected abnormalities. The spleen primordium develops normally in the absence of *Hox11* up until E13.5 but fails to expand thereafter (28). Why B-1a cells are essentially absent in *Hox11*−*/*− mice (3), remains unclear, although it has been proposed that this phenotype can be attributed to their asplenia. Indeed, transfer of *Hox11*-null FL cells into SCID mice reconstituted the B-1a compartment to normal levels, suggesting that defective B-1a cell generation in *Hox11*-null mice is not due to an intrinsic defect in B-1 cell progenitor populations (3). The *Hox11-*null mice could, however, have other unreported defects in supporting B-1 cell development or maintenance apart from absence of spleen. We, therefore, used another strategy to evaluate the requirement of spleen for B-1 cell development where we transferred “pre-splenic” E11 FL cells into splenectomized RAG1−/− mice. In this model, asplenia resulted only in a slight reduction in peritoneal B-1a cells rather than a complete absence of B-1a cells, as observed in Hox11−/− mice. Potential limitations of our model of asplenia are that FL cells were transferred into immunocompromised mice (RAG1−/−), for which lack of competing lymphocytes may compromise mechanisms that would otherwise work to control B-1a cell expansion. Although in one experiment we waited 30 days after splenectomy of RAG1−/− mice before FL cell transfer (Figure S3 in Supplementary Material), it is also possible that remnant spleen-derived factors would persist for this period of time and could have a supportive role in development of B-1 cells from the transferred FL cells. Finally, early transcription factors associated with spleen development are expressed already at embryonic age 11 days (28), and these may have been sufficient to initiate peritoneal B-1 cell development from the transferred E11 FL cells. Nonetheless, the spleen primordium is not generated before E12-13 (24) and since peritoneal B-1a cells were indeed generated from E11 FL transferred into splenectomized hosts, our study illustrates that an intact spleen is not unconditionally required for peritoneal B-1a cell development.

We demonstrated a slight reduction in B-1a cell frequencies, in the peritoneal cavity after splenectomy (Figures 2 and 3). Interestingly, in neonatally splenectomized mice, the frequencies of mature B cells in the bone marrow were increased at the expense of pre-B cells and B-1a cells (Figure 3). The reason for the altered immune cell composition in the bone marrow after splenectomy remains unclear and is a subject for further investigation. It was previously reported that bone marrow B-1a cells spontaneously secrete large quantities of IgM and thus are a main contributor to steady state serum IgM levels (31). We also demonstrated a reduction in bone marrow IgM antibody secreting cells in splenectomized mice. It is, therefore, possible that the reduction in bone marrow B-1a cells was at least partially responsible for reduced serum IgM in asplenic mice (29).

In summary, in the study reported here, we demonstrate that B-1a cells developed in the absence of an intact spleen, although a reduction in B-1a cell frequencies was observed when modeling asplenia from prenatal, neonatal, and adult stages of life.

## Ethics Statement

Studies were performed in accordance with institutionally approved protocols and with Committee for Animal Ethics (Stockholms Norra djurförsöksetiska nämnd) approval or Institutional Animal Care and Use Committee approval (IACUC).

## Author Contributions

GKP designed the study, performed experiments, and analyzed data and wrote the manuscript. XL, SK, and MÁ performed experiments and provided scientific input. BB designed the study and provided scientific input. GKH designed the study, provided scientific input, and wrote the manuscript. All authors have read and approved the manuscript.

## Conflict of Interest Statement

The authors declare that the research was conducted in the absence of any commercial or financial relationships that could be construed as a potential conflict of interest.

## Supplementary Material

Figure S1 Bone marrow B-1a cell numbers are decreased in splenectomized mice. Adult 12-week-old wild-type mice were splenectomized or sham-operated and 10 days later stained for **(A)** peritoneal cavity and **(B)** bone marrow B-1a and B-2 cells (defined as in Figure 1). Graphs display mean ± SD. Statistically significant differences are indicated by *, denoting *p* < 0.05 by unpaired *t*-test.

Figure S2 Representative stainings of bone marrow B-lineage populations. **(A)** Staining of bone marrow pro-B, (B220+CD43+CD19+), pre-B (B220+CD43−CD93+IgM−), immature B (B220+CD43−CD93+IgM+), and mature B (B220+CD43−CD93−IgM+) cells **(B)** Staining of mature bone marrow B-1a (CD93−CD19hiB220loCD43+CD5+) and B-2 (CD93−CD19+B220+/−) cells. Representative plots are from 6 weeks post neonatal splenectomy.

Figure S3 B-1a cell development from fetal liver cells transferred into already splenectomized hosts. Wild-type (wt) FL cells were isolated from 11 days old embryos (E11) and transferred to adult RAG1−/− mice that had undergone splenectomy or sham surgery 30 days earlier. At 6 weeks later the mice were sacrificed and analyzed for B-1a cells. Donor-derived B-1a cells were identified as IgM+Cd19hiB220loCD43+CD5+. No IgM positive cells were observed in the peritoneum of RAG1−/− mice that had not received wt cells (not shown). **(A)** Schematic of the study. **(B)** Representative plots of peritoneal cavity B cells after transfer of E11 cells into splenectomized versus sham-operated recipients. **(C)** Frequency of the indicated B cell subsets in the peritoneal cavity of recipient mice as gated from IgM+ (donor-derived cells). **(D)** Representative plots and frequencies of bone marrow B-1a and B-2 cells after transfer of E11 cells into splenectomized versus sham-operated recipients. Plots were gated from IgM+ cells (donor-derived cells). **(E)** Total IgM antibody levels at 6 weeks post fetal liver cell transfer. Sera were run in threefold dilution with a starting dilution of 1:10 and statistics calculated by comparing area under the curve. The experiment was performed once. Statistically significant differences are indicated by * denoting *p* < 0.05 by unpaired *t*-test.
