# Supplementary figures and images for: B-1a Cell Development in Splenectomized Neonatal Mice
Source: Front Immunol. 2018 Jul 30;9:1738. doi: 10.3389/fimmu.2018.01738 (PMC6077197; doi:10.3389/fimmu.2018.01738)

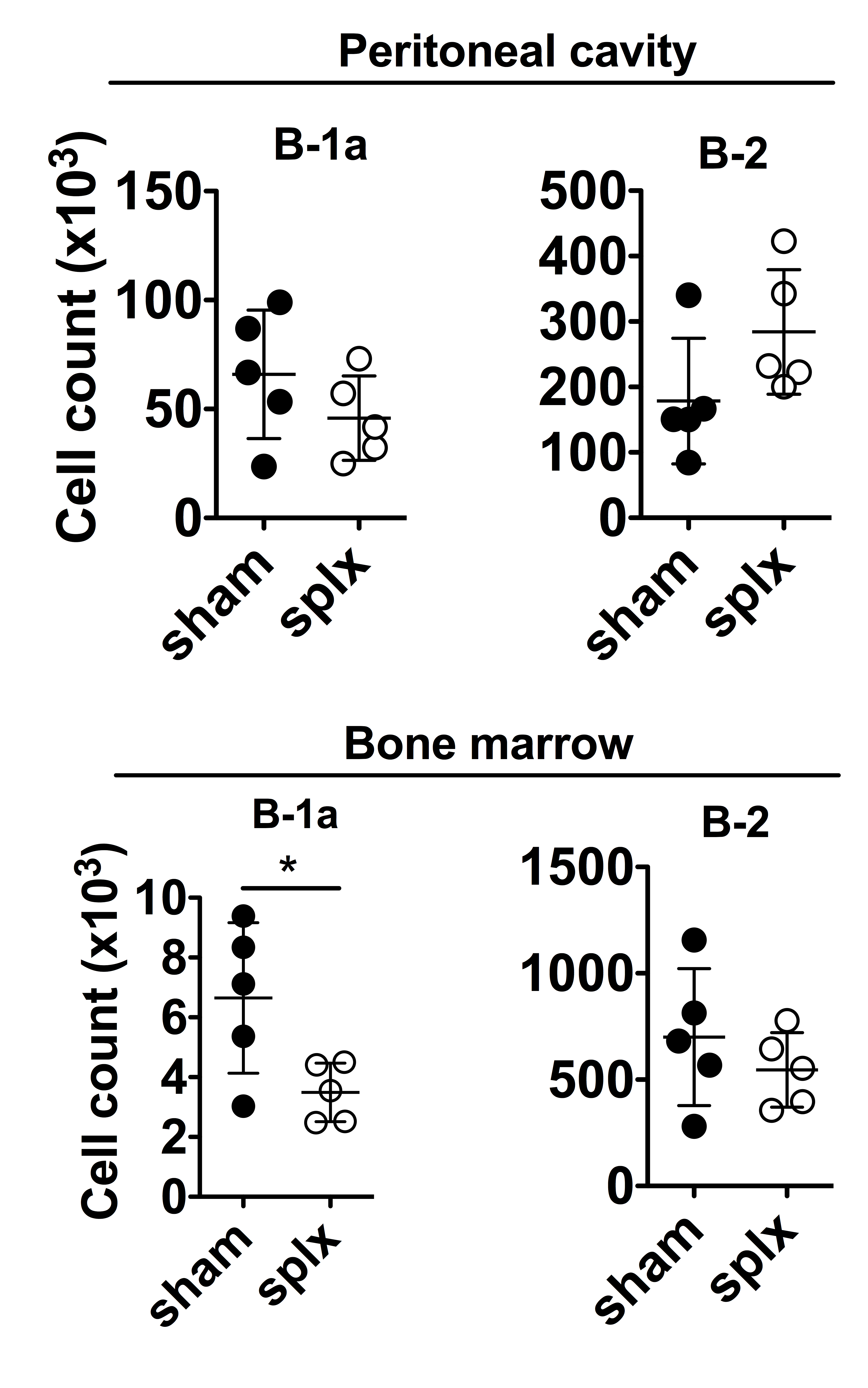

Supplement: Figure S1 — Bone marrow B-1a cell numbers are decreased in splenectomized mice. Adult 12-week-old wild-type mice were splenectomized or sham-operated and 10 days later stained for (A) peritoneal cavity and (B) bone marrow B-1a and B-2 cells (defined as in Figure 1). Graphs display mean ± SD. Statistically significant differences are indicated by *, denoting p < 0.05 by unpaired t-test. [file Image_1.tiff]

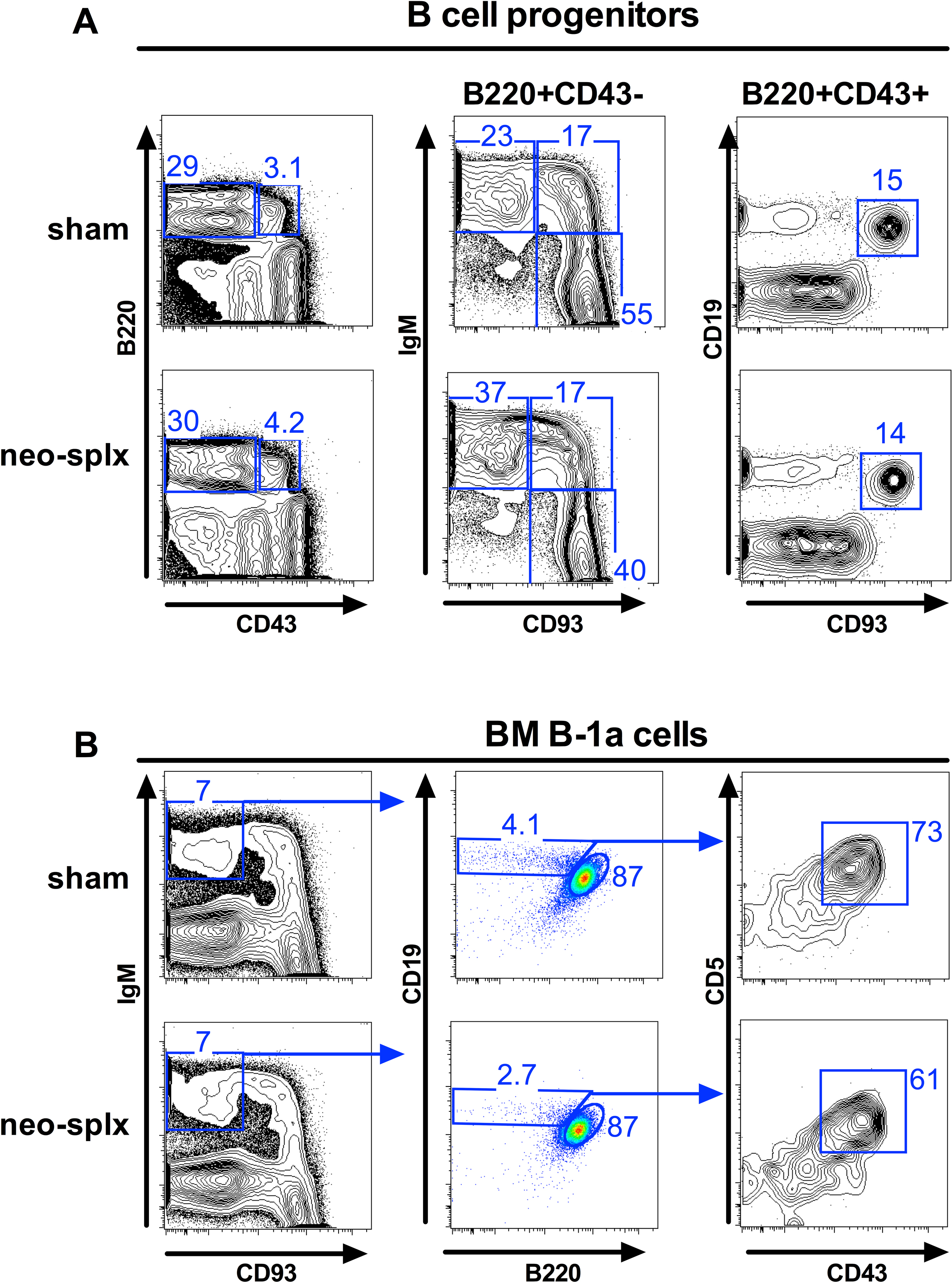

Supplement: Figure S2 — Representative stainings of bone marrow B-lineage populations. (A) Staining of bone marrow pro-B, (B220+CD43+CD19+), pre-B (B220+CD43−CD93+IgM−), immature B (B220+CD43−CD93+IgM+), and mature B (B220+CD43−CD93−IgM+) cells (B) Staining of mature bone marrow B-1a (CD93−CD19hiB220loCD43+CD5+) and B-2 (CD93−CD19+B220+/−) cells. Representative plots are from 6 weeks post neonatal splenectomy. [file Image_2.tif]

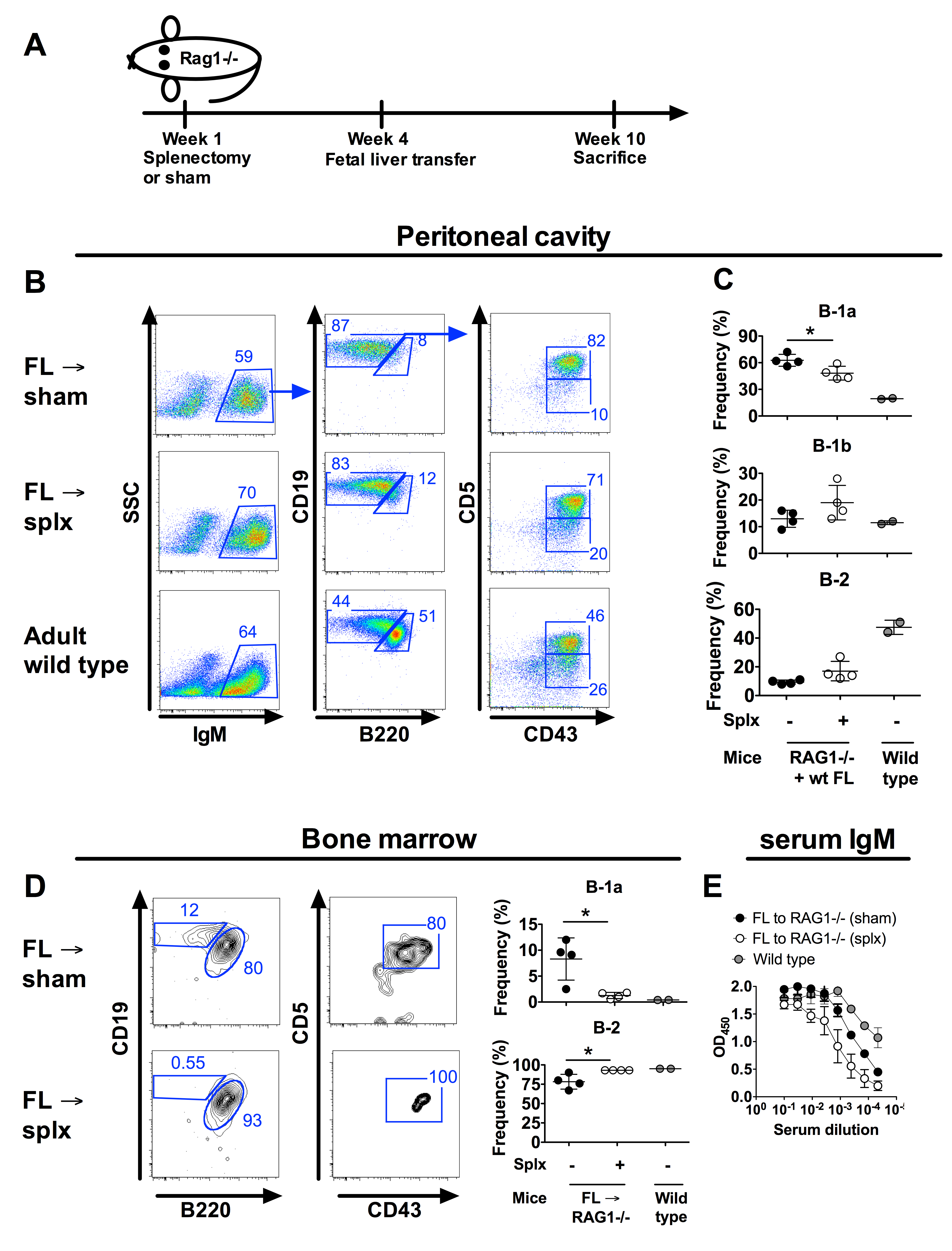

Supplement: Figure S3 — B-1a cell development from fetal liver cells transferred into already splenectomized hosts. Wild-type (wt) FL cells were isolated from 11 days old embryos (E11) and transferred to adult RAG1−/− mice that had undergone splenectomy or sham surgery 30 days earlier. At 6 weeks later the mice were sacrificed and analyzed for B-1a cells. Donor-derived B-1a cells were identified as IgM+Cd19hiB220loCD43+CD5+. No IgM positive cells were observed in the peritoneum of RAG1−/− mice that had not received wt cells (not shown). (A) Schematic of the study. (B) Representative plots of peritoneal cavity B cells after transfer of E11 cells into splenectomized versus sham-operated recipients. (C) Frequency of the indicated B cell subsets in the peritoneal cavity of recipient mice as gated from IgM+ (donor-derived cells). (D) Representative plots and frequencies of bone marrow B-1a and B-2 cells after transfer of E11 cells into splenectomized versus sham-operated recipients. Plots were gated from IgM+ cells (donor-derived cells). (E) Total IgM antibody levels at 6 weeks post fetal liver cell transfer. Sera were run in threefold dilution with a starting dilution of 1:10 and statistics calculated by comparing area under the curve. The experiment was performed once. Statistically significant differences are indicated by * denoting p < 0.05 by unpaired t-test. [file Image_3.tiff]
